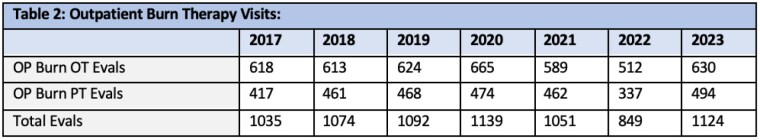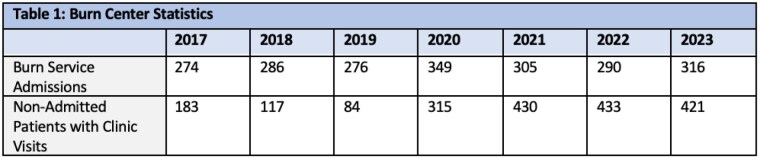# 803 Exploration of the Outpatient Site-of-Care Shift Within a Single Center

**DOI:** 10.1093/jbcr/iraf019.334

**Published:** 2025-04-01

**Authors:** Audrey O’Neil, Nicholas Kousagan, David Roggy, Brett Hartman

**Affiliations:** Richard M. Fairbanks Burn Center; Eskenazi Health; Richard M. Fairbanks Burn Center; Eskenazi Health

## Abstract

**Introduction:**

Nationally, site-of-care shifts, within the outpatient setting, are accelerating and expanding into more critical areas of healthcare. Outpatient procedures from joint replacements to cardiac procedures are becoming standards of care. However, burn care has been slower to respond. Despite advances in acute burn care increasing survival rates and shortening length of stay, challenges often limit feasibility of managing burn survivors solely within the outpatient setting, requiring inpatient admission. The purpose of this study was to review one center’s experience with the outpatient shift and explore the impact of a multidisciplinary team approach.

**Methods:**

A retrospective review was completed using the electronic medical record system (EMR) within this 15-bed adult verified burn center. The burn clinic staff includes a dedicated multidisciplinary team of nurse practitioners, nurses, medical assistants, physical therapy (PT), and occupational therapy (OT). A report was built to capture the Non-Admitted Patients with Outpatient Burn Clinic visits on a yearly basis and compared to inpatient Burn Service Admission rates between 2017-2023. Outpatient burn therapy evaluations for both PT and OT were also evaluated within the same time period, however EMR was unable to exclude patients with inpatient burn center admissions.

**Results:**

In the last 7 years, the burn center admission volume remained on average, 300 patients per year consistently. (Table1) The average number of non-admitted burn patients treated between 2017-2019 was 128 patients/year. However, in 2020, there was a significant shift in non-admitted patients treated in the outpatient clinic from 84 in 2019 to 315 in 2020. This number continued to increase over the last 3 years with 430 patients treated in 2021, 433 patients in 2022, and 421 patients in 2023.(Table 1) The resulting average/year of non-admitted burn patients between 2020-2023 was 271, increased by 53%. Burn outpatient therapy also saw on average 1052 patients per year over the last 7 years, but did not see an increase in volume compared to the burn clinic volumes (Table 2).

**Conclusions:**

In conclusion, it is feasible to conduct care within the outpatient setting for adult burn survivors. The burn center was able to avoid hospital acute admissions by providing multidisciplinary care within the outpatient setting. As a safety-net hospital, additional resources were present to assist patients overcome access barriers, however the multidisciplinary aspect of the clinic assisted in meeting all patient needs in a single visit.

**Applicability of Research to Practice:**

Structural changes in care delivery are necessary to accommodate the site of care shift, trending nationally in health care. Burn centers need to evaluate access to care within their centers and adapt to meet the need of burn survivors in a changing healthcare system.

**Funding for the Study:**

N/A